# Epidemiology of Functional Seizures Among Adults Treated at a University Hospital

**DOI:** 10.1001/jamanetworkopen.2020.27920

**Published:** 2020-12-29

**Authors:** Slavina B. Goleva, Allison M. Lake, Eric S. Torstenson, Kevin F. Haas, Lea K. Davis

**Affiliations:** 1 Division of Genetic Medicine, Department of Medicine, Vanderbilt University Medical Center, Nashville, Tennessee; 2 Vanderbilt Genetics Institute, Vanderbilt University Medical Center, Nashville, Tennessee; 3 Department of Molecular Physiology and Biophysics, Vanderbilt University, Nashville, Tennessee; 4 Division of Epidemiology, Department of Medicine, Vanderbilt University Medical Center, Nashville, Tennessee; 5 Vanderbilt Epidemiology Center, Institute for Medicine and Public Health, Vanderbilt University Medical Center, Nashville, Tennessee; 6 Department of Neurology, Vanderbilt University Medical Center, Nashville, Tennessee

## Abstract

**Importance:**

Functional seizures (formerly *psychogenic nonepileptic seizures*), paroxysmal episodes that are often similar to epileptic seizures in their clinical presentation and display no aberrant brain electrical patterns, are understudied. Patients experience a long diagnostic delay, few treatment modalities, a high rate of comorbidities, and significant stigma due to the lack of knowledge about functional seizures.

**Objective:**

To characterize the clinical epidemiology of a population of patients with functional seizures observed at Vanderbilt University Medical Center (VUMC).

**Design, Setting, and Participants:**

This case-control study included patients with functional seizures identified in the VUMC electronic health record (VUMC-EHR) system from October 1989 to October 2018. Patients with epilepsy were excluded from the study and all remaining patients in the VUMC medical center system were used as controls. In total, the study included 1431 patients diagnosed with functional seizures, 2251 with epilepsy and functional seizures, 4715 with epilepsy without functional seizures, and 502 200 control patients who received treatment at VUMC for a minimum of 3 years. Data were analyzed from November 2018 to March 2020.

**Exposure:**

Diagnosis of functional seizures, as identified from the VUMC-EHR system by an automated phenotyping algorithm that incorporated *International Classification of Diseases, Ninth Revision (ICD-9)* codes, *International Statistical Classification of Diseases and Related Health Problems, Tenth Revision (ICD-10)* codes, *Current Procedural Terminology* codes, and natural language processing.

**Main Outcomes and Measures:**

Associations of functional seizures with comorbidities and risk factors, measured in odds ratios (ORs).

**Results:**

Of 2 346 808 total patients in the VUMC-EHR aged 18 years or older, 3341 patients with functional seizures were identified (period prevalence, 0.14%), 1062 (74.2%) of whom were women and for which the median (interquartile range) age was 49.3 (39.4-59.9) years. This assessment replicated previously reported associations with psychiatric disorders including posttraumatic stress disorder (PTSD) (OR, 43.23; 95% CI, 34.47-54.22; P < 3.02 × 10^−5^), anxiety (OR, 11.43; 95% CI, 9.54-13.68; *P* < 3.02 × 10^−5^), and depression (OR, 11.10; 95% CI, 9.17-13.45; *P* < 3.02 × 10^−5^), and identified novel associations with cerebrovascular disease (OR, 4.00; 95% CI, 3.26-4.91; *P* < 3.02 × 10^−5^). An association was found between functional seizures and the known risk factor sexual assault trauma (OR, 10.26; 95% CI, 10.09-10.44; *P* < 3.02 × 10^−5^), and sexual assault trauma was found to mediate nearly a quarter of the association between female sex and functional seizures in the VUMC-EHR.

**Conclusions and Relevance:**

This case-control study found evidence to support previously reported associations, discovered new associations between functional seizures and PTSD, anxiety, and depression. An association between cerebrovascular disease and functional seizures was also found. Results suggested that sexual trauma may be a mediating factor in the association between female sex and functional seizures.

## Key Points

**Question:**

What is the period prevalence and comorbidity of patients with functional seizures?

**Findings:**

This case-control study including 3341 patients with functional seizures (period prevalence, 0.14%) identified from all patients at a university medical center found that posttraumatic stress disorder and sexual assault trauma were associated with functional seizures. Novel associations were identified, including cerebrovascular disease.

**Meaning:**

The findings of this study suggest that patients who present with seizures and additional psychiatric comorbidities, sexual assault trauma history, or cerebrovascular disease should be referred for video electroencephalogram diagnostic assessment.

## Teaser

This case-control study of patients at a large university medical center provides an epidemiological assessment of functional seizures and their association with comorbidities (eg, cerebrovascular disease) and risk factors (eg, sexual assault trauma).

## Introduction

Functional seizures (previously *psychogenic nonepileptic seizures*) are paroxysmal episodes that often include altered awareness or convulsions with presentation similar to epileptic seizures. The term *functional seizure* is favored because it (1) reduces stigma, (2) does not presuppose a specific etiology, and (3) fits with existing nosology of other functional neurological movement disorders.^1,2,3^ Functional seizures can be definitively distinguished from epileptic seizures based on video electroencephalogram (vEEG) recording of typical seizures, which are usually performed in an epilepsy monitoring unit.^4^ Underlying psychological factors are likely involved in the etiology of functional seizures. Once diagnosed, patients with functional seizures may be treated with various psychiatric therapeutic modalities, including cognitive behavioral therapy, but to date no medications are specifically approved for functional seizures.^5,6^ Functional seizures remains understudied, yet 20% to 30% of patients referred to epilepsy monitoring units are eventually diagnosed with functional seizures.^7^ Published studies include small sample sizes and limited epidemiological data.^8^ Given the challenging diagnosis, limited treatment options, and lack of awareness, there is a need for more research on functional seizures.^9,10,11^

The prevalence of functional seizures is estimated at 2 to 33 cases per 100 000 people (0.002%-0.033%)^12^ and approximately 17% to 22% of patients with functional seizures have concurrent epilepsy.^13,14,15^ Patients with functional seizures have a higher rate of psychiatric disorders (eg, posttraumatic stress disorder [PTSD], anxiety, depression) than both the general population and patients with epilepsy (risk ratio, 1.30; 95% CI = 1.14-1.48).^16^ Sexual assault trauma is a known risk factor for functional seizures, and it is thought that sexual assault, which also occurs more frequently among female individuals, could explain part of the female bias (ie, 73% women) in functional seizures.^10,17,18^ Epilepsy is also associated with increased psychiatric comorbidity, as well as increased risk for cerebrovascular disease. Little is known about whether similar risk for cerebrovascular disease exists in patients with functional seizures.^19,20,21,22,23^

Functional seizures are often, but not always, a diagnosis of clinical exclusion, and on average patients experience a diagnostic delay of 7 years.^13^ There are signs that can be used to positively distinguish functional seizures, such as a long duration of seizures, fluctuating course of seizures, and asynchronous movements.^24^ In the *Diagnostic and Statistical Manual of Mental Disorders* (Fifth Edition), functional seizures is classified under conversion disorder, subtype “with attacks or seizures,” and is defined as neurological symptoms that are incompatible with neuropathology. The absence of consistent nomenclature or specific *International Classification of Diseases* (*ICD*) classification hampers clinical recognition and treatment. Thus, a need remains for research to characterize the clinical manifestation of functional seizures, shorten the time to diagnosis, and advance treatments for patients with functional seizures.

This study characterizes the clinical epidemiology of functional seizures in a hospital population. We utilized a database of deidentified electronic health records (EHR) from Vanderbilt University Medical Center (VUMC-EHR). We addressed the research question through the (1) development of a clinically validated phenotyping algorithm to identify functional seizures cases, (2) estimation of the period prevalence of functional seizures in a hospital population, (3) systematic identification of comorbidities associated with functional seizures, and (4) investigation of the association between sexual assault trauma and functional seizures in a clinical population.

## Methods

### Sample and Data Description

The study population included all 2 346 808 unique VUMC patients from 1994 to 2019.^25^ Demographic characteristics, *International Classification of Diseases, Ninth Revision (ICD-9)* and *International Statistical Classification of Diseases and Related Health Problems, Tenth Revision (ICD-10)* codes, *Current Procedural Terminology* codes, and clinical notes were extracted from the medical record and mined for subsequent analyses. For the phenome-wide association study (PheWAS), we restricted the study population to adults (age ≥18 years) with at least 5 *ICD-9* or *ICD-10* codes on different days over at least 3 years, to exclude individuals who did not receive regular health care at VUMC and reduce bias caused by nonrandom missingness between cases and controls (ie, medical home) (eFigure 1 in the Supplement). A total of 341 patients with functional seizures did not meet criteria for the medical home definition and were not included in the PheWAS. Related ICD codes were organized into hierarchical code families (ie, “phecodes”) that represent broader categories of disease.^26^ This study was reviewed and approved by the VUMC institutional review board and exempted from informed consent requirements as non–human subjects research using deidentified medical data. This study followed the Strengthening the Reporting of Observational Studies in Epidemiology (STROBE) reporting guideline.

### Functional Seizures Case-Control Algorithm

**Figure 1.**
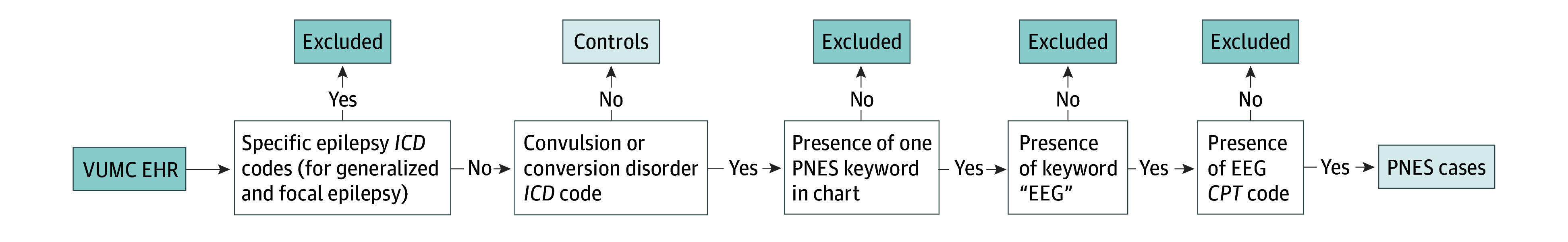
Algorithm for Detecting Functional Seizures Cases and Controls Within the VUMC-EHR All Vanderbilt University Medical Center electronic health records (VUMC-EHR) were initially included, then anyone with generalized or focal epilepsy *International Classification of Diseases, Ninth Revision (ICD-9)* and *International Statistical Classification of Diseases and Related Health Problems, Tenth Revision (ICD-10)* codes were excluded. Patients without convulsion or conversion disorder *ICD* codes were considered cases, while anyone with both a psychogenic nonepileptic seizures (PNES) keyword in their medical record and the presence of the keyword “EEG” (electroencephalogram) was included as a functional seizures case. *CPT* indicates *Current Procedural Terminology*.

Two separate functional seizures algorithms were developed under supervision of a clinical neurologist (K.F.H.) to reduce bias during algorithm development and medical record review. The primary algorithm was used to identify patients with functional seizures without concurrent epilepsy (Figure 1; eTable 1 in the Supplement). The secondary algorithm identified patients with functional seizures without exclusions for epilepsy (eAppendix 1, eFigure 2, and eTable 2 in the Supplement).^27^ The primary algorithm was validated by unmasked manual medical record review of 50 algorithm-identified cases (eAppendix 1 in the Supplement). Patients with sexual assault trauma were identified in the VUMC-EHR using regular expressions identification in clinical notes (eAppendix 1 in the Supplement).

### Statistical Analyses

#### Medical Record Review

Positive predictive value (PPV; PPV = total true cases / total algorithm-identified cases × 100) was calculated to determine the accuracy of the phenotyping approach for both functional seizures and sexual assault trauma. The records of patients identified were reviewed for documented evidence of a clinical diagnosis of functional seizures and classified as either (1) documented, clinically established, or probable functional seizures; or (2) possible functional seizures (criteria are further described in the eAppendix 1 in the Supplement).^24^ Additionally, mean and standard error of the reported time from seizure onset to functional seizures diagnosis were calculated using data extracted during medical record review.

#### Phenome-Wide Association Studies

PheWAS were performed to systematically assess the co-occurrence of functional seizures and all other phenotypes across the adult medical phenome after covariate adjustment. *ICD-9* codes were mapped to 1814 phecodes (ie, outcomes) as described and validated using a hierarchical grouping system by the Phecode Map version 1.2 project.^28^ For each of the 1653 phecodes, any individual with 2 or more phecode-mapped *ICD-9* codes was assigned case status for the corresponding phecode, while those with no phecode-mapped *ICD-9* codes were assigned as controls, and those with only 1 phecode-mapped *ICD-9* code were excluded from the analysis of that phecode. Individuals with related diagnosis codes, as determined by the Phecode Map version 1.2, were also excluded from controls due to an increased risk of false negatives due to misdiagnosis. *ICD-10* codes were not included as there is not yet a validated structure for *ICD-10* to phecode mapping. We required a minimum number of 100 cases for each phecode. We fitted 1653 logistic regressions to test the association between algorithm-defined binary functional seizures case/control status (predictor variable) and each phecode (outcome) after adjusting for potential mediators and confounders including sex, age (defined for each individual as median age across their medical record), density of records (in records per year), and EHR-reported race.^26,28^ PheWAS results were considered statistically significant if the *P* value of the association passed a Bonferroni corrected threshold for multiple testing (*P* < .05 / 1653 = 3.02 × 10^−5^).

#### Temporal Analysis Between Functional Seizures and Cerebrovascular Disease

After our PheWAS revealed an association between functional seizures and cerebrovascular disease, we performed additional analyses to further explore the temporal association between functional seizures (with epilepsy) diagnosis and coded cerebrovascular disease (eAppendix 1 in the Supplement). We determined whether cerebrovascular disease or functional seizures was more likely to occur in the EHR first, then whether there was any difference in the common comorbidities between those patients who presented with functional seizures first or cerebrovascular disease first. For patients with both functional seizures and cerebrovascular disease, we calculated the median (and interquartile range [IQR]) age at the first *ICD* code for cerebrovascular disease and the number of years from functional seizures diagnosis to first cerebrovascular disease code. We then binned patients into 3 groups: (1) functional seizures diagnosis more than 90 days before cerebrovascular disease, (2) functional seizures diagnosis within 90 days of cerebrovascular disease diagnosis, and (3) cerebrovascular disease diagnosis more than 90 days before functional seizures diagnosis. We compared these 3 groups of patients with functional seizures plus patients with cerebrovascular disease on 17 different clinical and demographic features (eTable 3 in the Supplement). We performed parallel analyses to examine the temporal association between cerebrovascular disease and epilepsy and to test for differences in the same 17 clinical features that were examined in functional seizures plus cerebrovascular disease cases.

#### Sexual Assault Trauma Mediation of Functional Seizures

We fitted a multivariable logistic regression model to adult functional seizures cases and controls to determine whether sexual assault trauma, female sex, or the interaction between them was associated with functional seizures case status. Additional covariates included median age of record, median body mass index, race/ethnicity, and record density. Next, we performed mediation analysis using the R mediation package (R Project for Statistical Computing) to test whether women were more likely to develop functional seizures because women are more likely to experience sexual assault trauma.

## Results

### Calculation of PPV From Automated EHR-Based Functional Seizures Phenotyping Algorithm

Thirty-five of 50 medical records (70%; 95% CI, 69.2%-70.8%) of patients with functional seizures reviewed were documented, clinically established, and probable functional seizures cases, while 14 records (28%; 95% CI, 27.21%-28.79%) were of possible functional seizures cases, and only 1 record (2%) was classified as “No functional seizures/Not enough information.” A medical student (A.M.L.) performed medical record review on 52 patients identified by the sexual assault trauma phenotyping approach and determined PPV was 90.4% (95% CI, 82.4%-98.4%).

### Medical Record Review to Determine the Mean Time From Seizure to Diagnosis

Thirty-two medical records of the 50 (64%) randomly selected for manual medical records review included written descriptions of patient-reported date of seizure onset. As a graduate student (S.B.G.) reviewed the medical records, she made note of the patient-reported date of seizure onset. Based on this data, the mean (SE) time from seizure onset to diagnosis of functional seizures was 6.6 (1.4) years.

#### Calculation of Functional Seizures Prevalence in a Clinical Population

The functional seizures algorithm identified 3341 patients older than 18 years with functional seizures in the VUMC-EHR, out of a total patient population of 2 346 808 (0.14%); 1062 (74.2%) of these patients were women, and their median (interquartile range) age was 49.3 (39.4-59.9) years. Of the total patient population, 752 024 (32.0%) VUMC patients met our definition for medical home patients; in the functional seizures case sample, 1431 (42.8%) met the definition for medical home (Table 1). The period prevalence of patients with functional seizures in the adult medical home population was 0.27% (1431 out of 523 593) and 1062 (74.2%) of the algorithm-identified functional seizures medical home patients were women (Table 1).

**Table 1.** Demographic Characteristics of Algorithm-Defined Functional Seizures Cases and Controls^a^

Characteristics	Patients, No. (%)	
	Controls (n = 502 200)	Functional Seizures Cases (n = 1431)
VUMC-EHR, No.	NA	3341
Medical home, No.	502 200	1431
Women	299 472 (59.6)	1062 (74.2)
Race		
Caucasian	417 523 (83.1)	1264 (88.3)
African American	56 549 (11.3)	166 (11.6)
Asian	7581 (1.5)	13 (0.9)
Unknown/other	18 097 (3.6)	30 (2.1)
Native American	1992 (0.4)	10 (0.7)
Ethnicity		
Hispanic/Latino(a)	9644 (1.9)	23 (1.6)
Unknown	19 176 (3.8)	37 (2.6)
Records per year, median (IQR)	6.03 (2.88-13.15)	9.07 (4.05-20.89)
Length of record, median (IQR), y	8.81 (5.56-12.99)	10.29 (6.26-14.68)
*ICD* codes, median (IQR), No.	49 (23-113)	80 (35-215.5)
Age, median (IQR), y	59.58 (45.02-72.48)	49.31 (39.40-59.87)
Median age of record, median (IQR), y	51 (36-65)	40 (30-51)

Abbreviations: EHR, electronic health records; *ICD*, *International Classification of Diseases*; IQR, interquartile range; VUMC, Vanderbilt University Medical Center.

^a^ Number of adult cases identified by functional seizures algorithm within the VUMC-EHR and how many of those meet a medical home definition, or those who have at least 5 *ICD* codes on different days over the span of at least 3 years.

#### Functional Seizures Comorbidity With Psychiatric and Neurological Disorder Codes

**Figure 2.**
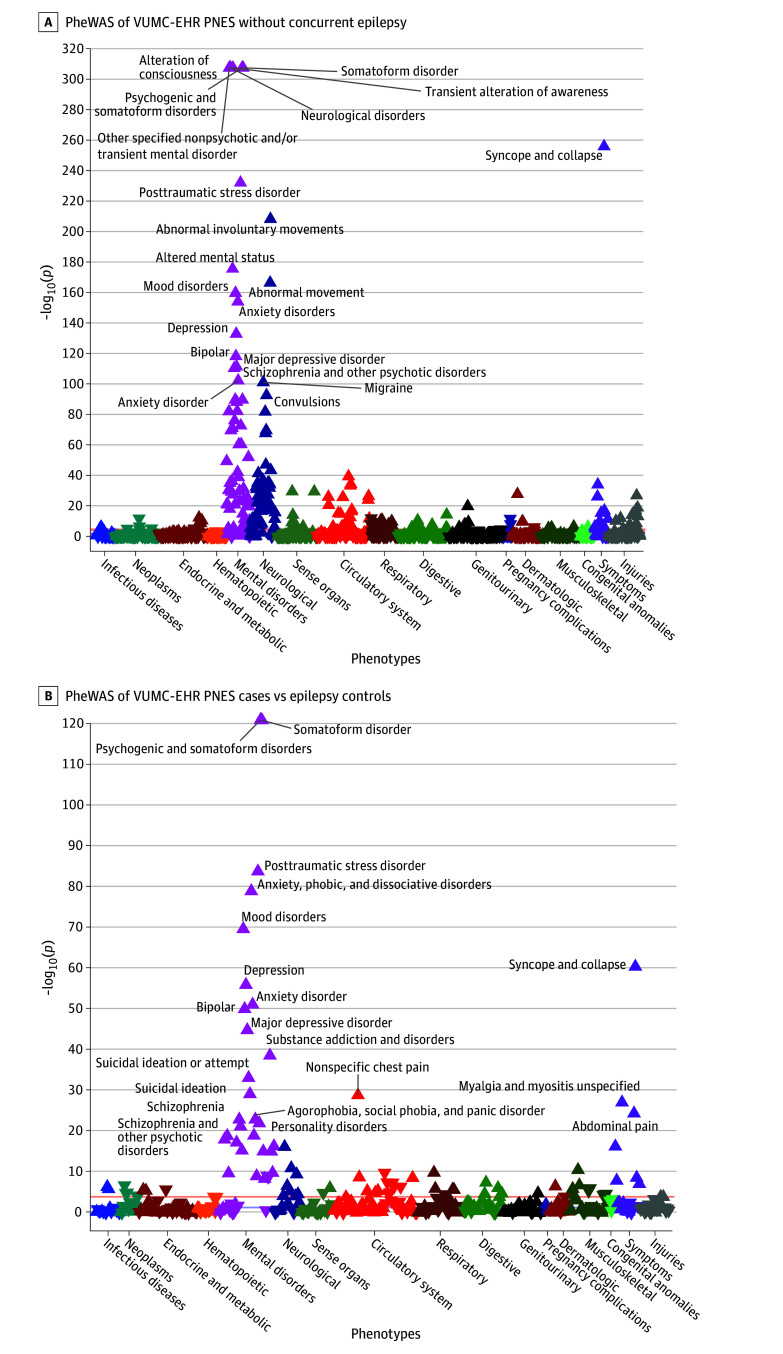
PheWAS of VUMC-EHR Functional Seizures Algorithm-Defined Cases vs Algorithm-Defined Controls and Epilepsy Controls Results are plotted by category of phenotypes, with each category shown in a different color. Only the top 20 associations are labeled to increase visibility of the graph. Upturned triangles represent positive associations with functional seizures case status, while downturned triangles represent negative associations with functional seizures case status. A horizontal blue line indicates the nominal *P* value level 0.05, while a horizontal orange line indicates the Bonferroni-corrected *P* value. PheWAS indicates phenome-wide association study; VUMC-EHR, Vanderbilt University Medical Center electonic health records.

Phenotypes most strongly associated with functional seizures when compared with algorithm-identified controls included psychogenic and somatoform disorders (OR, 365.52; 95% CI, 294.94-452.99; *P* < 3.02 × 10^−5^), somatoform disorder (OR, 799.14; 95% CI, 629.65-1014.25; *P* < 3.02 × 10^−5^), PTSD (OR, 43.23; 95% CI, 34.47-54.22; *P* < 3.02 × 10^−5^), mood disorders (OR, 11.83; 95% CI, 9.89-14.15; *P* < 3.02 × 10^−5^), and anxiety disorders (OR, 11.43; 95% CI, 9.54-13.68; *P* < 3.02 × 10^−5^) (Figure 2). We also identified associations with depression (OR, 11.10; 95% CI, 9.17-13.45; *P* < 3.02 × 10^−5^) and schizophrenia (OR, 24.58; 95% CI, 18.58-32.52, *P* < 3.02 × 10^−5^). Overall, 56 of 72 phecodes (77%) mapping to the mental disorders category were significantly associated with functional seizures after Bonferroni correction for multiple testing (*P* = 3.02 × 10^−5^). We found that 55 of 82 (67%) of the neurological disorder phecodes were significantly associated with functional seizures.

Table 2 includes the period prevalence of sexual assault trauma and 10 common psychiatric comorbidities among algorithm-defined functional seizures cases. These were 10 predominantly adult-afflicting psychiatric comorbidities for which *ICD* code definitions had previously been validated.^29^ The prevalence of most of these psychiatric comorbidities in the VUMC-EHR was similar to previously reported literature. However, prevalence of these psychiatric comorbidities was much higher in patients with functional seizures, ranging from 2.6% (phobia) to 30.2% (major depressive disorder).

**Table 2.** Psychiatric Phenotypes and Sexual Assault Trauma Comorbidities in Functional Seizures Cases

Phenotype	No. of cases total (No. comorbid with Functional Seizures)^a^	VUMC-EHR MH prevalence, %	Prevalence within patients with functional seizures, %	Ratio of prevalence within functional seizures:VUMC to EHR MH
Schizophrenia	10 322 (176)	1.97	12.30	6.24
Major depression	36 069 (432)	6.89	30.19	4.38
Bipolar disorder	14 789 (254)	2.82	17.75	6.28
Insomnia	32 493 (195)	6.21	13.63	2.20
Posttraumatic stress disorder	8088 (339)	1.54	23.69	15.34
Obsessive-compulsive disorder	2086 (39)	0.40	2.73	6.84
Alcohol abuse	13 630 (105)	2.60	7.34	2.82
Alcohol use—long-term	5164 (46)	0.99	3.21	3.26
Phobia	2550 (37)	0.49	2.59	5.31
Anxiety	38 898 (391)	7.43	27.32	3.68
Sexual assault trauma	4357 (188)	0.83	13.14	15.79
Functional seizures	1431	0.27	NA	NA

Abbreviations: EHR, electronic health records; MH, medical home; VUMC, Vanderbilt University Medical Center.

^a^ The total number of adult patients in the SD medical home was 523 584.

### Functional Seizures Co-occurrence With Cerebrovascular Disease

We identified 13 novel and significant associations between cerebrovascular disease codes and functional seizures. These included cerebrovascular disease (OR, 4.00; 95% CI, 3.26-4.91; *P* < 3.02 × 10^−5^) and transient cerebral ischemia (OR, 5.39; 95% CI, 4.13-7.03, *P* < 3.02 × 10^−5^) (Table 3). Analyses with PheWAS among functional seizures cases vs epilepsy cases (Figure 2) and epilepsy cases vs controls (eFigure 3 in the Supplement) showed that cerebrovascular disease is broadly associated with epileptic and nonepileptic seizures. Cerebrovascular disease (phecode 433) was more strongly associated with epilepsy than functional seizures (functional seizures: OR, 5.39; 95% CI, 4.13-7.03; *P* = 2.6 × 10^−40^; epilepsy: OR, 6.41; 95% CI, 5.80-7.09; *P* < 3.02 × 10^−05^).

**Table 3.** Functional Seizures–Associated Cerebrovascular Disease Phenotypes

Phecode	Phecode description	OR (95% CI)	*P* value	No.	
				Cases	Controls
433	Cerebrovascular disease	4.00 (3.26-4.91)	2.57 × 10^−40^	19 161	374 563
433.31	Transient cerebral ischemia	5.39 (4.13-7.03)	2.80 × 10^−35^	7475	374 563
433.3	Cerebral ischemia	5.19 (3.98-6.76)	3.24 × 10^−34^	7886	374 563
433.8	Late effects of cerebrovascular disease	4.89 (3.42-6.98)	2.56 × 10^−18^	3510	374 563
433.6	Acute, but ill-defined cerebrovascular disease	6.04 (3.98-9.16)	2.60 × 10^−17^	2350	374 563
433.2	Occlusion of cerebral arteries	3.41 (2.47-4.69)	6.77 × 10^−14^	6680	374 563
433.21	Cerebral artery occlusion, with cerebral infarction	3.42 (2.47-4.74)	1.72 × 10^−13^	6476	374 563
430	Intracranial hemorrhage	3.91 (2.63-5.80)	1.37 × 10^−11^	3088	374 563
433.5	Cerebral aneurysm	4.19 (2.54-6.93)	2.22 × 10^−8^	1618	374 563
430.1	Subarachnoid hemorrhage	4.00 (3.26-4.91)	5.68 × 10^−8^	1187	374 563
430.2	Intracerebral hemorrhage	3.68 (2.09-6.47)	6.20 × 10^−6^	1525	374 563
430.3	Subdural hemorrhage	5.33 (2.48-11.46)	1.80 × 10^−5^	782	374 563
433.1	Occlusion and stenosis of precerebral arteries	2.54 (1.64-3.92)	2.86 × 10^−5^	6741	374 563

Abbreviation: OR, odds ratio.

Among patients diagnosed with both functional seizures and cerebrovascular disease, 29% were diagnosed with functional seizures prior to the onset of cerebrovascular disease, 23% were diagnosed with cerebrovascular disease and functional seizures within 90 days of each other, and 48% were diagnosed with functional seizures after cerebrovascular disease (eFigure 4, eTable 3 in the Supplement). A parallel analysis of epilepsy and cerebrovascular disease revealed similar patterns (eTable 4 in the Supplement).

### Functional Seizures Association With Sexual Assault Trauma

We identified a total of 10 036 individuals in the EHR (0.36%) who met inclusion criteria for sexual assault trauma, including 1853 men and 8183 women (eTable 5 in the Supplement). Among these patients, 4357 also met criteria for the medical home population and were over the age of 18. Of patients reporting sexual assault, 188 (4.3%) met criteria for functional seizures. Conversely, the prevalence of sexual assault trauma among patients with functional seizures was 13.1%, 15.8 times more prevalent than in the general medical home population. The prevalence of sexual assault trauma among patients with epilepsy was 132 out of 4715 (2.8%).

Using a multivariable logistic regression, we found that sexual assault trauma was significantly associated with functional seizures (OR, 10.26, 95% CI, 10.09-10.44; *P* < 3.02 × 10^−05^) (eTable 6 in the Supplement) after adjusting for age, sex, median body mass index, race, and medical record density. While our analysis showed that female sex was also significantly associated with functional seizures (vs male: OR, 0.64; 95% CI, 0.51-0.77; *P* < 3.02 × 10^−05^), there was no significant interaction between sex and sexual assault trauma on functional seizures diagnosis (OR, 1.30; 95% CI, 0.80-1.81; *P* = .31). Sexual assault trauma mediated 22% of the variance in functional seizures diagnosis associated with female sex.

## Discussion

This study aimed to examine clinical and epidemiological characteristics of functional seizures. First, we identified patients with functional seizures in the VUMC-EHR by developing an automated phenotyping algorithm with a PPV of 98%. The complete algorithm for identification of functional seizures cases is provided in eAppendix 1 in the Supplement and deposited in Phenotype KnowledgeBase for future research use by others (Figure 1; eTable 1 in the Supplement). Medical record review of these patients also revealed that the mean (SE) time from first seizure to functional seizures diagnosis in these patients was 6.6 (1.4) years. Based on the number of patients identified by our algorithm in proportion to the total number of patients in our hospital system, we calculated the period prevalence of functional seizures to be 0.14% in our clinical population. When restricting to the medical home population, the period prevalence was 0.27%. In analyzing comorbidities among patients with functional seizures, we found evidence supporting previous reports that functional seizures co-occurs with psychiatric and neurological disorders, and that patients with functional seizures are nearly 16 times more likely than the average hospital patient to have a documented history of sexual assault trauma. Moreover, we found that sexual assault trauma explains 22% of the increased rate of functional seizures in women. Finally, we also discovered that functional seizures co-occurs with cerebrovascular disease at a rate higher than expected by chance.

In a 2000 study, Benbadis and Hauser^12^ estimated functional seizures to occur in approximately 2 to 33 people per 100 000 (0.002%-0.033%). The directly calculated period prevalence of functional seizures in the VUMC clinical population was, as expected, much higher at 3341 per 2 346 808 (0.14%). VUMC is home to an epilepsy monitoring unit, which increases the number of patients with functional seizures relative to the general population. Thus, the prevalence in the VUMC EHR may not be generalizable. However, it is critical to understand the prevalence of functional seizures in a medical center setting for multiple reasons. First, efforts aimed at improving clinical care for patients with functional seizures are bolstered by awareness of the frequency of functional seizures in a clinical setting. Second, the prevalence in a clinical setting also provides motivation for development of an ICD diagnostic classification specific to functional seizures. The visibility issues that patients with functional seizures face was further substantiated by our medical record review which indicated that the average time from first seizure to functional seizures diagnosis was 6.6 years, closely matching older reports.^13^

Our PheWAS results suggest that patients with functional seizures are at risk for additional chronic health conditions including cerebrovascular disease. While associations between cerebrovascular disease and epilepsy are widely reported, no robust associations between functional seizures and cerebrovascular disease have been reported so far to our knowledge.^19,20,21,22,23^ Diagnoses of cerebral ischemia, occlusion of cerebral arteries, and intracranial hemorrhage were all significantly associated with functional seizures in our data (Table 3). These results are consistent with a previous report detailing functional seizures and comorbid chronic medical conditions.^30^ However, we observed no clear illness trajectory from functional seizures to cerebrovascular disease, and in fact found that cerebrovascular disease often preceded the onset of functional seizures (eFigure 4 and 5, eTable 3 in the Supplement). Moreover, we found no difference in the rate of functional seizures risk factors or comorbidities between those patients who experienced functional seizures first compared with those who experienced cerebrovascular disease first. The finding that cerebrovascular disease may precede functional seizures could be explained by brain trauma or psychological distress related to cerebrovascular disease, paralleling prior data demonstrating that stroke is a risk factor for later-onset epilepsy. These findings have important implications for the management of patients who develop poststroke seizures. Specifically, diagnostic vEEG evaluation for poststroke seizures is critical to confirm the diagnosis of epilepsy and/or functional seizures.

The top associations in our analysis were somatoform disorder (OR, 799.14; 95% CI, 629.65-1014.25) and psychogenic and somatoform disorders (OR, 365.52; 95% CI, 294.94-452.99), which was expected as ICD codes used the FS phenotyping algorithm are also present within these phecode categories. Consistent with previous reports, we found functional seizures is associated with multiple psychiatric and neurological disorders compared with the general hospital population and patients with epilepsy (Figure 2; eFigure 3 in the Supplement). These multiple illnesses may be causally linked to functional seizures etiology or pathophysiology. Alternatively, multiple diagnoses may accumulate for a patient with functional seizures while undergoing clinical treatment and care since functional seizures has a broad differential diagnosis. Therefore, further research is needed to clarify if any causal relationship exists between functional seizures and the numerous diagnoses with which the current study identified associations. Given that patients with functional seizures are diagnosed with a mean (SE) 4.75 (0.13) different psychiatric diagnoses (compared with 2.6 [0.054] in patients with epilepsy and 0.85 [0.003] in hospital controls), we suggest a clinical guideline: that patients experiencing seizures with a high burden of psychiatric illness be considered for a functional seizures diagnosis and referred for diagnostic vEEG monitoring. This is especially important as early functional seizures diagnosis and treatment are associated with better outcome.^31,32,33^ Overall, we believe that this novel EHR-based study provides important rationale and motivation for ongoing EHR-based research to improve the complex and challenging clinical care of patients with functional seizures.

Functional seizures have a much higher prevalence in women. Consistent with previous reports, approximately 74% of functional seizures cases in our cohort were in women.^9,17,34,35,36^ Previous studies also indicate that women with functional seizures were 8 times more likely to report sexual assault trauma than men with functional seizures.^17^ We found that sexual assault trauma and PTSD were approximately 16 times more frequent in patients with functional seizures compared with the general hospital population (Table 2). Among patients with functional seizures, 15.8% of women and 5.42% of men reported a history of sexual assault trauma. While women with functional seizures reported more sexual assault trauma than men with functional seizures, this reflected the increased overall rate of sexual assault among women (1.08%) compared with men (0.25%). Indeed, given sexual assault trauma exposure, there was no significant difference between men and women in the rate of functional seizures diagnosis, indicating that men and women who experience sexual assault trauma are at equivalent risk of developing functional seizures (eTable 6 in the Supplement). Furthermore, results from the mediation analysis indicate that the overall increased rate of sexual assault trauma among women actually explains nearly a quarter of the increased rate of functional seizures among women.

### Limitations

This study has several limitations. As previously noted, the medical center used as setting in this study is home to an epilepsy monitoring unit, which may make the prevalence of patients with functional seizures at VUMC not generalizable to the general population. Second, EHRs are not comprehensive of all health care utilization and may have missing data. To help with this problem, we restricted all patients to a medical home definition (at least 5 ICD codes on different days over the span of at least 3 years). Third, we were not able to individually review every patient’s medical record identified by our algorithm to confirm diagnosis.

## Conclusions

In this study, we identified novel associations between functional seizures and cerebrovascular disease, estimated that approximately 22% of the female bias in functional seizures is mediated by the occurrence of sexual assault trauma, and replicated previous associations between functional seizures and many psychiatric and neurological phenotypes. Taken together, these findings provide evidence for the hypothesis that functional seizures, while influenced by multiple complex factors exhibiting interindividual differences, may be considered a physical manifestation of the neurological damage caused by trauma.

## Notes

Supplement. **eAppendix 1.** Methods **eAppendix 2.** Results **eTable 1.** Criteria for Algorithm to Identify Functional Seizures Patients, Excluding Those Who Have Concurrent Epilepsy **eTable 2.** Criteria for Algorithm to Identify Functional Seizures Patients Regardless of Epilepsy Status **eTable 3.** Demographics for 92 Patients Who Were Diagnosed With Both Cerebrovascular Disease and Functional Seizures **eTable 4.** Demographics for 735 Patients Who Were Diagnosed With Both Cerebrovascular Disease and Epilepsy **eTable 5.** Criteria to Identify Sexual Assault Trauma (SAT) Patients **eTable 6.** Functional Seizures Case Status and Sex Are Significantly Associated With Sexual Assault in the VUMC-EHR **eTable 7.** Body Mass Index information for Functional Seizures and Epilepsy Cases as Compared to Controls **eFigure 1.** Functional Seizures Patients Have Longer, More Dense Medical Records as Compared to Controls **eFigure 2.** Flow Chart of Algorithm Used to Identify Functional Seizures Patients Irrespective of Their Epilepsy Case/Control Status **eFigure 3.** PheWAS of VUMC EHR ICD-Code Defined Epilepsy Cases Versus Controls **eFigure 4.** Histogram of the Number of Years from Date of Functional Seizures Diagnosis to Cerebrovascular Diagnosis in 92 Patients Who Had Both CVD and Functional Seizures **eFigure 5.** Temporal Analysis of the Development of Functional Seizures and Cerebrovascular Disease in 92 Patients **eFigure 6.** Functional Seizures Patients Have Greater BMIs Than the Control Group and Epilepsy Patients
