# Errors in Abstract, Results, and Table

**DOI:** 10.1001/jamanetworkopen.2026.28030

**Published:** 2026-07-21

In the Original Investigation titled “Epidemiology of Functional Seizures Among Adults Treated at a University Hospital,”^1^ published December 29, 2020, the odds ratios reported in the Abstract, Results, and Table 3 were incorrect. An error in the code used to generate the odds ratios resulted in a substantial underestimation of the point estimates. The authors have corrected the code and rerun the relevant analyses. The revised values do not change the direction of the associations or the overall conclusions. The article has been corrected,^1^ and the authors have explained how the error occurred in a Comment.^2^
