# Supplementary material for: Epidemiology of Functional Seizures Among Adults Treated at a University Hospital
Source: JAMA Netw Open. 2020 Dec 29;3(12):e2027920. doi: 10.1001/jamanetworkopen.2020.27920 (PMC7772716; doi:10.1001/jamanetworkopen.2020.27920)
Supplement: Supplement. — eAppendix 1. Methods eAppendix 2. Results eTable 1. Criteria for Algorithm to Identify Functional Seizures Patients, Excluding Those Who Have Concurrent Epilepsy eTable 2. Criteria for Algorithm to Identify Functional Seizures Patients Regardless of Epilepsy Status eTable 3. Demographics for 92 Patients Who Were Diagnosed With Both Cerebrovascular Disease and Functional Seizures eTable 4. Demographics for 735 Patients Who Were Diagnosed With Both Cerebrovascular Disease and Epilepsy eTable 5. Criteria to Identify Sexual Assault Trauma (SAT) Patients eTable 6. Functional Seizures Case Status and Sex Are Significantly Associated With Sexual Assault in the VUMC-EHR eTable 7. Body Mass Index information for Functional Seizures and Epilepsy Cases as Compared to Controls eFigure 1. Functional Seizures Patients Have Longer, More Dense Medical Records as Compared to Controls eFigure 2. Flow Chart of Algorithm Used to Identify Functional Seizures Patients Irrespective of Their Epilepsy Case/Control Status eFigure 3. PheWAS of VUMC EHR ICD-Code Defined Epilepsy Cases Versus Controls eFigure 4. Histogram of the Number of Years from Date of Functional Seizures Diagnosis to Cerebrovascular Diagnosis in 92 Patients Who Had Both CVD and Functional Seizures eFigure 5. Temporal Analysis of the Development of Functional Seizures and Cerebrovascular Disease in 92 Patients eFigure 6. Functional Seizures Patients Have Greater BMIs Than the Control Group and Epilepsy Patients [file jamanetwopen-e2027920-s001.pdf]

Supplemental Online Content

Goleva SB, Lake AM, Torstenson ES, Haas KF, Davis LK. Epidemiology of functional seizures among adults treated at a university hospital. *JAMA Netw Open*. 2020;3(12):e2027920. doi:10.1001/jamanetworkopen.2020.27920

eAppendix 1. Methods
eAppendix 2. Results
eTable 1. Criteria for Algorithm to Identify Functional Seizures Patients, Excluding Those Who Have Concurrent Epilepsy
eTable 2. Criteria for Algorithm to Identify Functional Seizures Patients Regardless of Epilepsy Status
eTable 3. Demographics for 92 Patients Who Were Diagnosed With Both Cerebrovascular Disease and Functional Seizures
eTable 4. Demographics for 735 Patients Who Were Diagnosed With Both Cerebrovascular Disease and Epilepsy
eTable 5. Criteria to Identify Sexual Assault Trauma (SAT) Patients
eTable 6. Functional Seizures Case Status and Sex Are Significantly Associated With Sexual Assault in the VUMC-EHR
eTable 7. Body Mass Index information for Functional Seizures and Epilepsy Cases as Compared to Controls
eFigure 1. Functional Seizures Patients Have Longer, More Dense Medical Records as Compared to Controls
eFigure 2. Flow Chart of Algorithm Used to Identify Functional Seizures Patients Irrespective of Their Epilepsy Case/Control Status
eFigure 3. PheWAS of VUMC EHR ICD-Code Defined Epilepsy Cases Versus Controls
eFigure 4. Histogram of the Number of Years from Date of Functional Seizures Diagnosis to Cerebrovascular Diagnosis in 92 Patients Who Had Both CVD and Functional Seizures
eFigure 5. Temporal Analysis of the Development of Functional Seizures and Cerebrovascular Disease in 92 Patients
eFigure 6. Functional Seizures Patients Have Greater Bmis Than the Control Group and Epilepsy Patients

This supplemental material has been provided by the authors to give readers additional information about their work.

© 2020 Goleva SB et al. *JAMA Network Open.*

eAppendix 1. Methods

1. **Functional Seizures case/control algorithm development**

Both algorithms were created by filtering criteria joined with Boolean operators. First, we required one or more convulsion or conversion disorder (ICD9 codes 300.11 OR 780.39 OR ICD10 codes R56.9 OR F44.5)[1] AND the presence of regular expressions indicating functional seizures-related keywords in charts ("psychogenic nonepileptic," "pseudoseizure," "psychogenic seizure," "nonepileptic seizure", or " pnes "). Although functional seizures can present as syncopal events, we have not included this phrase in our algorithm because in patient charts such as the ones we mined, this would be a nonspecific symptom and would add noise to our algorithm. Next we required the presence of both the keyword "EEG" AND one or more EEG CPT code (Group 95812-95830: Routine Electroencephalography (EEG) Procedures and Group 95950-95967: Special EEG Testing Procedures) to ensure that patients had an EEG performed at VUMC. Eighty-two percent of potential functional seizures cases with an "EEG" keyword also had an EEG procedure CPT code present in their chart.

2. **Chart Review**

Fifty charts were randomly selected from the functional seizures algorithm identified cases and were reviewed by one rater (SBG), who was trained by a clinical neurologist (KFH).

The charts were reviewed for positive clinical diagnosis of functional seizures based on a publication by LaFrance et al describing the minimum requirements for the diagnosis of functional seizures.[2] Positive predictive value (PPV) was calculated for two groups based on this paper: (a) documented, clinically established, or probable functional seizures; and (b) possible functional seizures. Based on this paper, the first criteria for a positive diagnosis of documented, clinically established, or probable functional seizures was defined as having chart history characteristics consistent with functional seizures. The second criteria was having a seizure witnessed by a clinician who either 1) reviewed the video EEG recording and found the semiology typical of functional seizures, or 2) is experienced in diagnosis of seizure disorders and found the seizure to show semiology typical of functional seizures, regardless of whether or not it was on vEEG. The third criteria was that there was no epileptiform activity in routine or sleep-deprived interictal EEG, in routine or ambulatory ictal EEG during a typical event, or immediately before, during or after ictus captured on ictal video EEG. A positive diagnosis for possible functional seizures was defined as having a history consistent with functional seizures, event matched functional seizures semiology by witness report or self-report/description, and that there was no epileptiform activity in routine or sleep-deprived interictal EEG.$f11$ KFH was consulted as needed to adjudicate.

Prior to review of these 50 charts, SG reviewed 100 charts, and KH also reviewed 37 of these charts. The goal of this first round of chart review was to train the reviewer (SG) to identify key phrases and language used by VUMC neurologists to interpret video EEG and to confirm diagnoses. SG, KH, and LKD sat in conference for approximately 4 hours to discuss the levels of evidence observed and the certainty of diagnosis given video EEG results.

For the sexual assault trauma analysis, AL manually reviewed 52 charts to identify the presence of confirmatory statements describing a history or current experience of sexual assault trauma.

Standard error (SE) and confidence intervals (CI) for the positive predictive value of each algorithm was calculated using the following formulas, where TP = number true positive, FP = number false positive:

$$SE = \sqrt{\frac{PPV * (1 - PPV)}{TP + FP}}$$

$$CI = PPV \pm 1.96 * SE$$

3. **Extraction of sexual assault trauma from the EHR**

While it is likely to be under-reported at the time of the assault, a history of sexual assault can be extracted from the medical record. Based on an initial review of 25 charts, which were randomly selected, we identified eight inclusion phrases (i.e., "his/her sexual assault", "history of sexual abuse", "history of rape", etc.) and four exclusion phrases

(i.e., “denies history of sexual abuse”, “no hx of sexual assault”, etc.) to identify cases (Supplementary Table 5). Although ICD codes do exist for sexual assault trauma, they were infrequently used. The primary mode of identification of patients who experienced sexual assault trauma in the EHR was through the identification of regular expressions (e.g., “history of sexual abuse”, “was raped”) within clinical notes.

Because sex is randomly determined at conception, the assumption of random assignment to the “treatment” condition holds, thus the patient sex (male/female) was coded as the “treatment” variable. Sexual assault trauma was then coded as the mediator and functional seizures as the outcome in a model-based mediation analysis.[3] We used 1,000 bootstrap comparisons to determine empirical confidence intervals and determine statistical significance of the mediation model. Additionally, we ran the mediation analysis with four different randomly generated seeds to ensure that the results and empirical p-values of the analysis remained stable across all permutations.

4. Development of Medical Home Population

The total study population used for algorithm development and prevalence calculations included the entire hospital population (N = 2,346,808). However, the phenome-wide association study was restricted to a subset of this total population called the “medical home” population. The medical home is a heuristic definition that restricts the total sample to a subpopulation with more complete medical record data to facilitate the comparison of comorbidity patterns between cases and controls. The definition applied required the presence of at least 5 codes over a period of at least 3 years. The reason for this restriction is to ameliorate the impact of missing data in controls which can result in a biased upward estimate in the regression coefficients in a case vs. control comparison in the PheWAS.

5. PheWAS of Function seizures compared to epilepsy cases

We compared algorithm identified functional seizures cases (N = 1,431) to epilepsy cases (4,715). Epilepsy cases for this analysis were defined as those that had at least three ICD codes for either generalized (GE) (FE) or focal epilepsy as previously defined.[4] Finally, we repeated the analysis a final time and compared epilepsy cases (4,175) to epilepsy controls (n = 496,890). Epilepsy controls were defined as all other patients, excluding functional seizures cases, functional seizures patients meeting exclusion criteria for functional seizures, and any patients with either one or two GE or FE ICD codes.

6. Functional seizures comorbidity sex-differences analysis

We performed a phenome-wide functional seizures by sex interaction analysis to determine whether comorbidities were more common among males or females with functional seizures after accounting for any baseline sex-difference in the prevalence of the comorbidity. Phenotypes that exceeded a Bonferroni corrected interaction p-value ($p < 3.02E-05$) demonstrated a significant diagnosis by sex interaction.

7. Functional seizures date of diagnosis analyses

For each patient identified as a functional seizures case, we established the date of the first clinical suspicion of functional seizures, defined as the first mention of a functional seizures keyword in clinical notes. We used the date of EEG administration (with a functional seizures keyword appearing in the patient’s chart within 30 days) to define the date of the diagnosis of functional seizures. We then determined the average time from the first mention to the diagnosis for algorithm defined functional seizures cases. Furthermore, during the manual chart review we documented patient-reported onset of seizures.

8. Cerebrovascular disease identification

Date of diagnosis of cerebrovascular disease was identified using the date of the first ICD code of a list of ICD9 codes that were found to be significantly associated with functional seizures and epilepsy (all of which overlapped). The ICD9 code list was as follows: 430, 433, 433.8, 430.2, 433.2, 433.21, 433.3, 433.31, 430.3, 433.6, 430.1, 433.1, 433.5, 433.11, 433.12.

9. Calculation of BMI from the EHR

© 2020 Goleva SB et al. *JAMA Network Open.*

Body mass index (BMI) data was obtained from the EHR. All individuals had multiple BMI measurements from various visits to the VUMC. BMI in the EHR is prone to recording errors.[5] To remove erroneous BMI records, we first z-score scaled the longitudinal BMI measurements collected within each individual, then removed any measurement with a Z-score below -3 or above 3. Next, we calculated median BMI value for each individual.

Using the cleaned median BMI values for each individual with available data, we calculated the mean and SE of BMI for functional seizures cases without epilepsy as defined by our algorithm (n = 1,605), for functional seizures cases with epilepsy as defined by our second algorithm (n = 1,058), and for controls (n = 488,398) as defined by our algorithm. We also calculated the mean and SE BMI value for epileptic patients (n = 6,186) for comparison and replication of prior studies.[6] Epileptic patients were defined as anyone who had either an ICD10 code from group G40 or an ICD9 code from group 345 for either generalized or focal epilepsy, as previously reported.[1,4] We used the R package ggplot2 for plotting, and a one-way ANOVA was performed in R followed by a Tukey post-hoc analysis to determine statistical significance of the difference in BMI between each group.

It is also worth noting that while all of our analyses were performed separately with and without median BMI included as a covariate. However, results for analyses excluding the median BMI covariate are not shown as they were very similar to the analyses including median BMI as a covariate.

© 2020 Goleva SB et al. *JAMA Network Open.*

eAppendix 2. Results

1. **EEG keyword presence in charts and CPT code presence are strongly correlated**

Across the entire VUMC-EHR, 66,936 patients had an EEG CPT code, 109,523 patients had “EEG” written in their charts, and 62,249 patients had both an EEG CPT code and keyword (93% of patients with an EEG CPT code and 57% of patients with an EEG keyword).

2. **Mental, neurological, and cerebrovascular disorders are associated with epilepsy**

We also performed a PheWAS analysis between epilepsy cases and epilepsy controls, which showed 47 out of 71 mental disorder codes were significantly associated with epilepsy, as well as 60 out of 82 neurological Phecodes (Supplementary Figure 3). We also saw that 35 associations with circulatory system codes were significant, several of which were among the highest associations with epilepsy.

Additionally, PheWAS comparing comorbidity patterns between functional seizures cases and epilepsy cases showed that 72% of the psychiatric, 42% of the neurological and 20% of the circulatory system phenotypes that were associated with functional seizures, remained associated with functional seizures even when the comparison group was limited to epilepsy cases (Figure 1B).

3. **Comorbidity patterns for functional seizures do not differ by sex**

There were no significant differences between males and females in the prevalence of each functional seizures comorbidity.

4. **BMI is significantly higher in functional seizures patients than controls**

One-way ANOVA and a post-hoc Tukey test showed that functional seizures cases without concurrent epilepsy had significantly higher median BMI measurement than algorithm-defined controls (include result here) or epilepsy patients (include result here) (Supplementary Figure 6). Additionally, a greater proportion of algorithm-defined functional seizures cases without concurrent epilepsy were considered obese (BMI>30) (39%) compared to algorithm-defined controls (33%), epileptic patients (27%), and functional seizures patients with concurrent epilepsy (37%)(Supplementary Table 7).

© 2020 Goleva SB et al. *JAMA Network Open.*

eTable 1. Criteria for algorithm to identify functional seizures patients, excluding those who have concurrent epilepsy

Functional seizures inclusion keywords (>= 1)	Inclusion ICD codes (>= 1)	Inclusion CPT codes (>= 1)	Additional inclusion criteria	Exclusion Epilepsy ICD codes in group (>= 1)
"Psychogenic non-epileptic" *(or nonepileptic or non epileptic)*	300.11 – conversion disorder	Group 95812-95830: Routine Electroencephalography (EEG) Procedures	Genotyping data available	345.1
"Pseudoseizure"	780.39 – other convulsions	Group 95950-95967: Special EEG Testing Procedures	Presence of keyword "EEG" in chart	345.10
"Psychogenic seizure"	R56.9 – unspecified convulsions	..	..	345.4
"Non-epileptic seizure" *(or nonepileptic or non epileptic)*	F44.5 – conversion disorder with seizures or convulsions	..	..	345.40
" PNES "	..	..	..	345.5
..	..	..	..	345.50
..	..	..	..	G40.20
..	..	..	..	G40.30
..	..	..	..	G40.00
..	..	..	..	G40.10

Inclusion ICD codes, inclusion keywords and other criteria, and exclusion ICD codes are all listed.

© 2020 Goleva SB et al. *JAMA Network Open.*

eTable 2. Criteria for algorithm to identify functional seizures patients regardless of epilepsy status

Functional seizures inclusion keywords (>= 1)	Inclusion ICD codes (>= 1)	Inclusion CPT codes (>= 1)	Additional inclusion criteria
"Psychogenic non-epileptic" *(or nonepileptic or non epileptic)*	300.11 – conversion disorder	Group 95812-95830: Routine Electroence-phalography (EEG) Procedures	Genotyping data available
"Pseudoseizure"	780.39 – other convulsions	Group 95950-95967: Special EEG Testing Procedures	Presence of keyword "EEG" in chart
"Psychogenic seizure"	R56.9 – unspecified convulsions	..	..
"Non-epileptic seizure" *(or nonepileptic or non epileptic)*	F44.5 – conversion disorder with seizures or convulsions	..	..
" PNES "	..	..	..

Inclusion ICD codes, inclusion keywords and other inclusion criteria are all listed.

© 2020 Goleva SB et al. *JAMA Network Open.*

eTable 3. Demographics for 92 patients who were diagnosed with both cerebrovascular disease and functional seizures

	**CVD and functional seizures codes (n = 92)**	**Functional seizures before CVD (n = 27)**	**CVD before functional seizures (n = 44)**	**Functional seizures and CVD simultaneous (n = 21)**
Age at first cerebrovascular code, Median (Q1 – Q3)	49.78 (39.03 – 55.51)	45.27 (37.29 – 54.79)	50.09 (41.83 – 55.68)	51.27 (38.87 – 58.58)
Age at functional seizures suspicion, Median (Q1 – Q3)	49.03 (38.10 – 57.30)	38.80 (32.95 – 49.10)	53.79 (43.46 – 60.66)	51.30 (38.68 – 58.34)
Age at functional seizures diagnosis, Median (Q1 – Q3)	49.71 (38.41 – 57.82)	38.80 (32.95 – 49.14)	53.79 (45.08 - 61.35)	51.30 (38.68 – 58.58)
Years from cerebrovascular code to functional seizures diagnosis, Median (Q1—Q3)	0.19 (-0.85 - 3.48)	-3.98 (-6.86 to -1.54)	3.89 (1.40 - 5.77)	0.01 (0.00 - 0.07)
PTSD comorbidity, N (%)	20 (22)	6 (22)	10 (23)	4 (19)
SAT comorbidity, N (%)	14 (15)	6 (22)	5 (11)	3 (14)
Migraine comorbidity, N (%)	27 (29)	7 (26)	14 (32)	6 (29)
Schizophrenia comorbidity, N (%)	3 (3)	1 (4)	1 (2)	1 (5)
MDD comorbidity, N (%)	20 (22)	5 (19)	11 (25)	4 (19)
Bipolar disorder comorbidity, N (%)	10 (11)	3 (11)	6 (14)	1 (5)
Insomnia comorbidity, N (%)	9 (10)	2 (7)	4 (9)	3 (14)
PTSD comorbidity, N (%)	0 (0)	0 (0)	0 (0)	0 (0)
Anxiety comorbidity, N (%)	29 (32)	6 (22)	17 (39)	6 (29)
Phobia comorbidity, N (%)	14 (15)	5 (19)	6 (14)	3 (14)
Alcoholism comorbidity, N (%)	5 (5)	2 (7)	2 (5)	1 (5)
OCD comorbidity, N (%)	5 (5)	2 (7)	2 (5)	1 (5)
Female sex, N (%)	64 (70)	17 (63)	33 (75)	14 (67)
Race, N (%)				
Caucasian	81 (88)	23 (85)	38 (86)	20 (95)
African American	11 (12)	4 (15)	6 (14)	1 (5)
Ethnicity, N (%)				
Hispanic/Latino(a)	2 (2)	1 (4)	0 (0)	1 (5)
Median BMI, Median (Q1 – Q3)	28.91 (24.72 – 35.61)	28.27 (24.13 – 32.03)	28.31 (24.03 – 36.12)	32.86 (26.57 – 37.34)
Records per year, Median (Q1 – Q3)	19.26 (8.61 – 61.02)	23.54 (6.79 – 48.33)	26.46 (9.65 – 68.30)	11.52 (7.85 – 34.07)
Length of record, Median (Q1 – Q3)	11.09 (7.38 - 15.75)	12.00 (8.80 - 15.38)	11.11 (7.80 - 15.99)	10.62 (7.13 - 11.85)
Number of ICD codes, Median (Q1 – Q3)	241.50 (68.25 – 648)	212 (67 – 607)	409.5 (102 – 819.5)	100 (56 – 399)
Age, Median (Q1 – Q3)	59.14 (48.94 – 65.91)	52.35 (42.72 – 60.94)	62.85 (53.47 – 68.04)	60.99 (53.35 – 65.84)
Median Age of Record, Median (Q1 – Q3)	51.50 (40 – 59)	46 (37.25 – 55)	54.50 (43.75 – 60.25)	51 (42 – 58)
Cardiovascular subcategory ICD codes (% of total CVD ICD codes)				
Cerebral hemorrhage	12	17	9	10
Cerebral artery occlusion	19	23	18	16
Cerebral ischemia	29	23	33	30
Cerebrovascular disease	35	30	37	41
Cerebral aneurysm	4	5	4	1
Cerebral atherosclerosis	1	2	0	1

Demographics are also shown for three subgroups of these patients: those who developed functional seizures before CVD (n = 27); those who developed functional seizures and CVD within three months of each other (n = 21); and those who developed CVD before functional seizures (n = 44). The proportion of males and females, race, ethnicity, density of records, median age across the medical record, age at first CVD code, age at functional seizures suspicion, age at functional seizures diagnosis, and percentages of CVD subcategories represented in each group. ICD codes for cerebral hemorrhage included 430, 430.2, 430.3, and 430.1. ICD codes for cerebral artery occlusion included 433.2, 433.21, 433.1, and 433.11. ICD codes for cerebral ischemia included 433.3 and 433.31. ICD codes for cerebrovascular diseases included 433, 433.8, and 433.6. The ICD code for cerebral aneurysm was 433.5, and the ICD code for cerebral atherosclerosis was 433.12.

© 2020 Goleva SB et al. *JAMA Network Open.*

eTable 4. Demographics for 735 patients who were diagnosed with both cerebrovascular disease and epilepsy

	**CVD and Epi codes (n = 735)**	**Epi before CVD (n = 208)**	**CVD before Epi (n = 374)**	**Epi and CVD simultaneous (n = 153)**
Age at first cerebrovascular code, Median (Q1 – Q3)	55.27 (42.72 – 65.18)	52.31 (40.03 – 61.81)	54.31 (43.62 – 65.07)	60.96 (48.26 – 70.49)
Age at first epilepsy code, Median (Q1 – Q3)	55.74 (42.07 - 66.16)	46.07 (33.68 – 57.73)	58.70 (45.91 – 68.55)	61.02 (48.30 – 70.49)
Days from epilepsy code to cerebrovascular, Median (Q1—Q3)	-106 (-1006.5 to 289)	1195 (607.5 – 2739.8)	-990 (-2089.8 to -350.2)	-1 (-28 to 0)
SAT, N (%)	10 (1)	3 (1)	4 (1)	3 (2)
PTSD, N (%)	18 (2)	9 (4)	7 (2)	2 (1)
Female sex, N (%)	357 (49)	104 (50)	183 (49)	70 (46)
Race, N (%)				
Caucasian	599 (81)	180 (87)	293 (78)	126 (82)
African American	127 (17)	25 (12)	77 (21)	25 (16)
Asian	6 (1)	2 (1)	3 (1)	1 (1)
Unknown/Other	1 (0)	0 (0)	1 (0)	0 (0)
Ethnicity, N (%)				
Hispanic/Latino(a)	14 (2)	4 (2)	7 (2)	3 (2)
Median BMI, Median (Q1 – Q3)	27.45 (23.69 – 31.84)	27.14 (23.50 – 31.51)	27.45 (24.03 – 32)	27.75 (23.14 – 31.16)
Records per year, Median (Q1 – Q3)	29.15 (14.40 – 56.46)	24.07 (11.25 – 44.43)	35.03 (17.22 – 64.94)	26.28 (13.67 – 53.67)
Length of record, Median (Q1 – Q3)	12.56 (8.05 – 16.79)	14.06 (9.50 – 17.45)	13.16 (8.48 – 16.84)	9.21 (6.12 – 14.12)
Number of ICD codes, Median (Q1 – Q3)	339 (158 – 676)	288 (142 – 556.8)	413.5 (181.2 – 791.2)	250 (148 – 535)
Age, Median (Q1 – Q3)	66.4 (54.4 – 75.72)	61.32 (48.52 – 71.63)	67.60 (56.01 – 78.25)	71.14 (59.35 – 77.81)
Median Age of Record, Median (Q1 – Q3)	58 (46 – 68)	52.50 (40.00 – 62.00)	59 (48 – 70)	63 (50 – 70)
Cardiovascular subcategory ICD codes (% of total CVD ICD codes)				
Cerebral hemorrhage	20	19	18	27
Cerebral artery occlusion	25	25	26	22
Cerebral ischemia	20	20	21	18
Cerebrovascular disease	33	33	32	32
Cerebral aneurysm	2	3	2	2
Cerebral atherosclerosis	1	1	1	1

Demographics are also shown for three subgroups of these patients: those who developed Epilepsy before CVD (n = 208); those who developed Epilepsy and CVD within three months of each other (n = 153); and those who developed CVD before Epilepsy (n = 374). The proportion of males and females, race, ethnicity, density of records, median ages across the medical record, age at first CVD code, age at first generalized or focal epilepsy code, and percentages of CVD subcategories are shown for each group. ICD codes for cerebral hemorrhage included 430, 430.2, 430.3, and 430.1. ICD codes for cerebral artery occlusion included 433.2, 433.21, 433.1, and 433.11. ICD codes for cerebral ischemia included 433.3 and 433.31. ICD codes for cerebrovascular diseases included 433, 433.8, and 433.6. The ICD code for cerebral aneurysm was 433.5, and the ICD code for cerebral atherosclerosis was 433.12.

© 2020 Goleva SB et al. *JAMA Network Open.*

eTable 5. Criteria to identify sexual assault trauma patients

Include	Exclude
history (OR hx OR h/o) of sexual abuse	no history (OR hx OR h/o) of sexual abuse
history (OR hx OR h/o) of sexual assault	denies history (OR hx OR h/o) of sexual abuse
sexual (OR sexually) abuse(d) by	no history (OR hx OR h/o) of sexual assault
reports (OR reported) a rape	denies history (OR hx OR h/o) of sexual assault
her (OR his) rape	
was raped	
sexually abused him (OR her)	
secondary to rape (OR sexual abuse OR sexual assault)	

Patients were exclusively identified using natural language processing of patients' clinical notes using various inclusion phrases and exclusion phrases. The inclusion phrases and exclusion phrases used are both reported here.

© 2020 Goleva SB et al. *JAMA Network Open.*

eTable 6. Functional seizures case status and sex are significantly associated with sexual assault in the VUMC-EHR

Variable	Pr(>|z|)	OR	CI
Sexual Assault and Trauma	5.36E-146	10.26	10.09-10.44
Sex (Male)	4.04E-11	0.64	0.51-0.77
SAT:Sex	0.31	1.30	0.80-1.81

A multivariable logistic regression functional seizures ~ SAT + sex + SAT*sex was performed to determine whether functional seizures case/control status, sex, or the interaction of functional seizures case status were correlated with sexual assault case status. P-values, odds ratio (OR) and the 95% confidence interval (CI) are presented for each variable. The multivariable logistic regression used included these variables and additional covariates for median age of record, median BMI, race, and the density of records.

© 2020 Goleva SB et al. *JAMA Network Open.*

eTable 7. Body Mass Index information for functional seizures and epilepsy cases as compared to controls

Phenotype	Sample size	Mean body mass index (SE)	Obese (% BMI over 30)
Control	488,398	28.17 (0.01)	33.46
Epilepsy	6,186	26.29 (0.09)	26.59
Functional seizures only	1,605	29.01 (0.20)	39.31
Functional seizures and epilepsy	1,058	28.64 (0.24)	37.43

Average and SE of median BMI are shown for controls as identified by our algorithm, epilepsy patients as identified by ICD codes, functional seizures cases as identified by our algorithm, and functional seizures cases with epilepsy as identified by our second algorithm. All participants analyzed had a median age of records over 18. The sample size and percentage of patients from each group that were considered obese (BMI over 30) is also shown.

© 2020 Goleva SB et al. *JAMA Network Open.*

eFigure 1. Functional seizures patients have longer, more dense medical records as compared to controls

A. B.

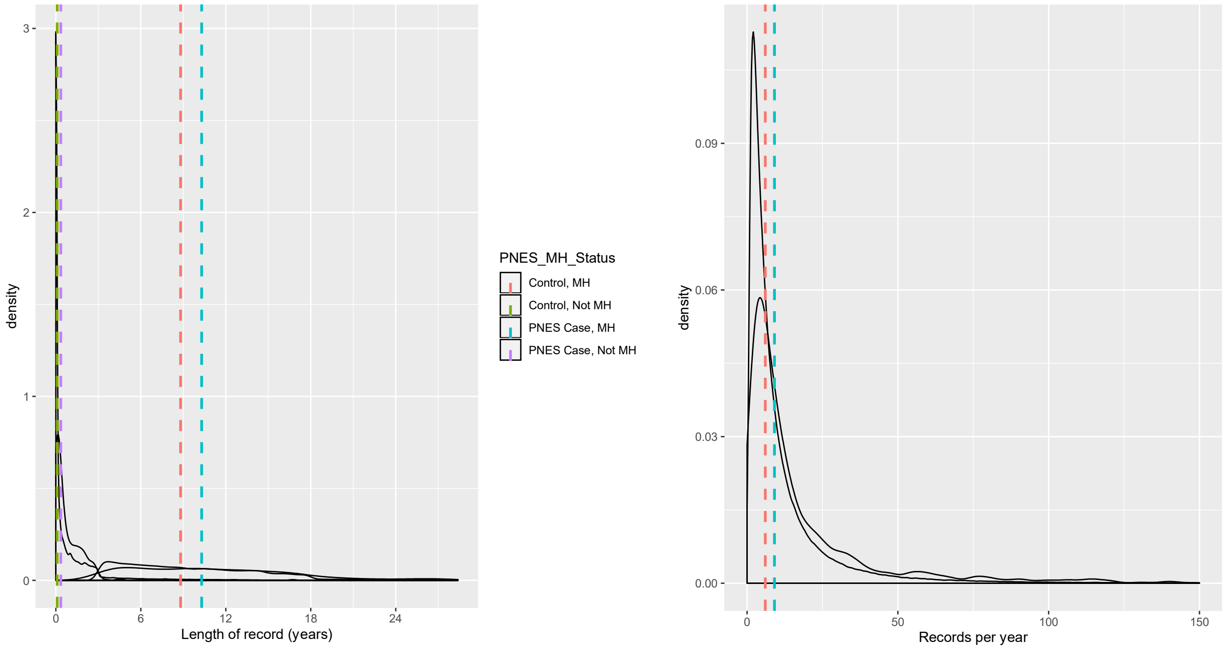

A. Density histogram of length of record is plotted in years for functional seizures cases (blue, purple) versus controls (red, green) in both medical home (red, blue) and outside of it (green, purple). Additionally, the median value for each group is plotted as a vertical dashed line. B. Density histogram of records per year in functional seizures cases (blue) and controls (red) within the medical home. Median values are plotted as vertical dashed line for both groups.

© 2020 Goleva SB et al. *JAMA Network Open.*

eFigure 2. Flow chart of algorithm used to identify functional seizures patients irrespective of their epilepsy case/control status

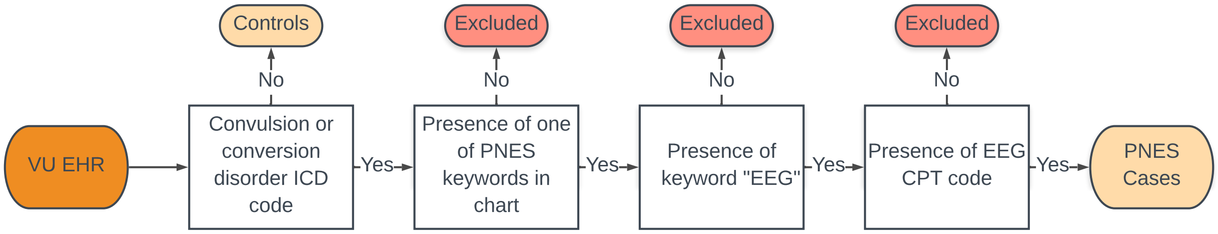

eFigure 3. PheWAS of VUMC EHR ICD-code defined epilepsy cases versus controls.

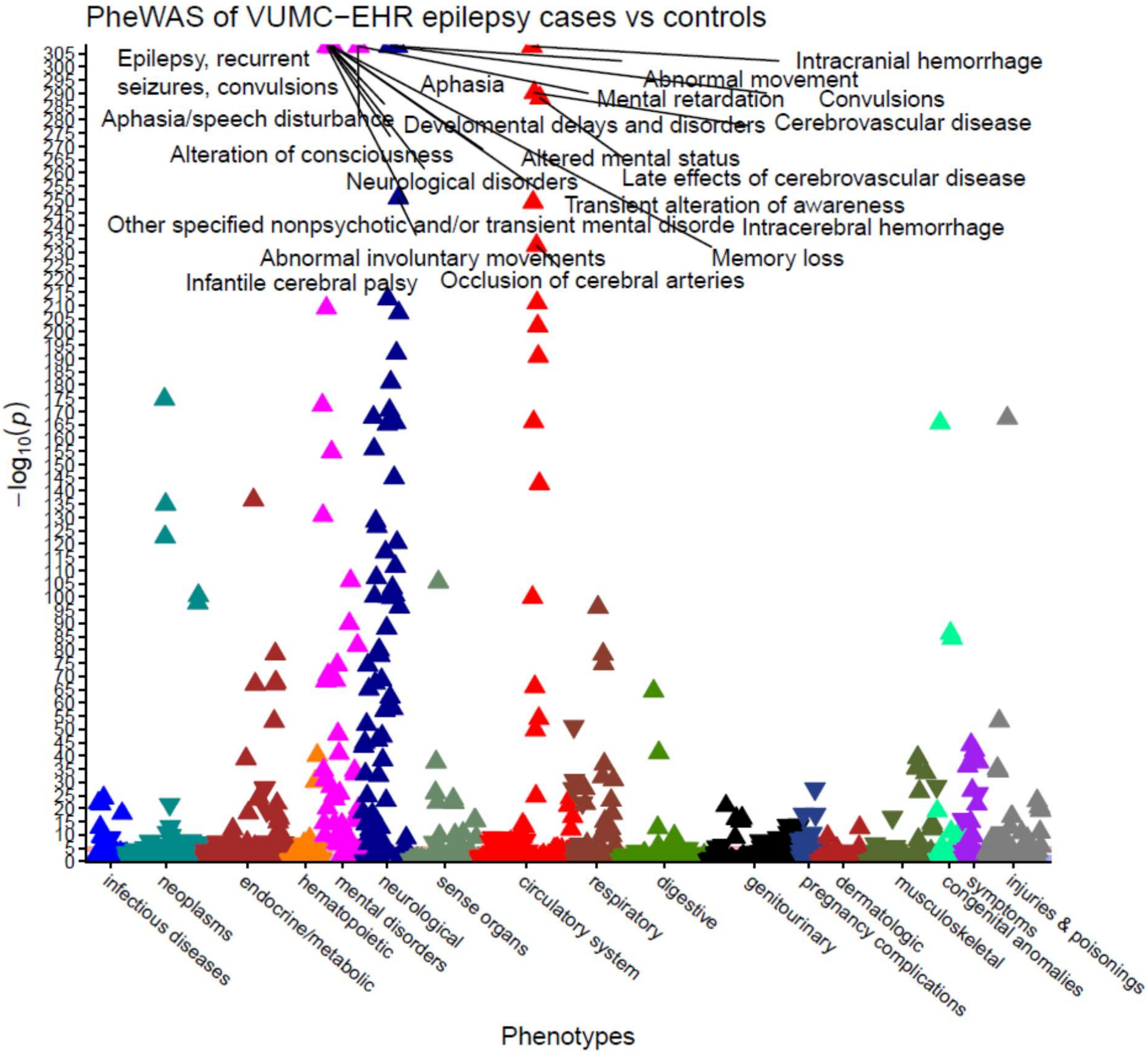

Results are plotted by category of phenotypes, with each category shown in a different color. Only the top 20 associations are labeled to increase visibility of the graph. Additionally, upturned triangles represent positive associations with functional seizures case status, while downturned triangles represent negative associations with functional seizures case status. A horizontal blue line indicates the nominal p-value level 0.05, while a horizontal red line indicates the Bonferroni corrected p-value.

© 2020 Goleva SB et al. *JAMA Network Open.*

eFigure 4. Histogram of the number of years from date of functional seizures diagnosis to cerebrovascular diagnosis in 92 patients who had both CVD and functional seizures

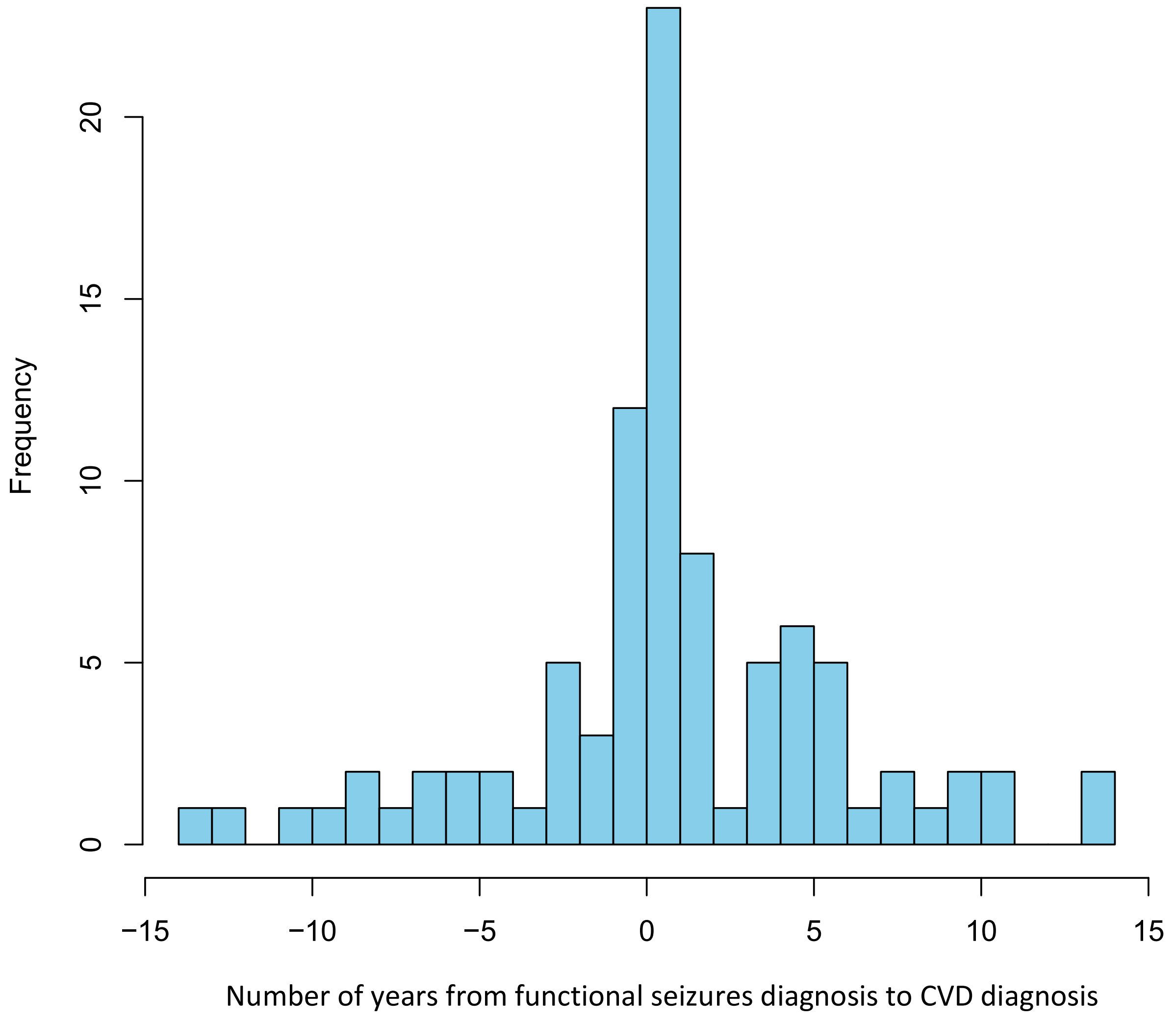

Negative values indicate patients who were diagnosed with CVD before functional seizures, while positive values indicate patients who were diagnosed with functional seizures before CVD.

© 2020 Goleva SB et al. *JAMA Network Open.*

eFigure 5. Temporal analysis of the development of functional seizures and cerebrovascular disease in 92 patients

A.

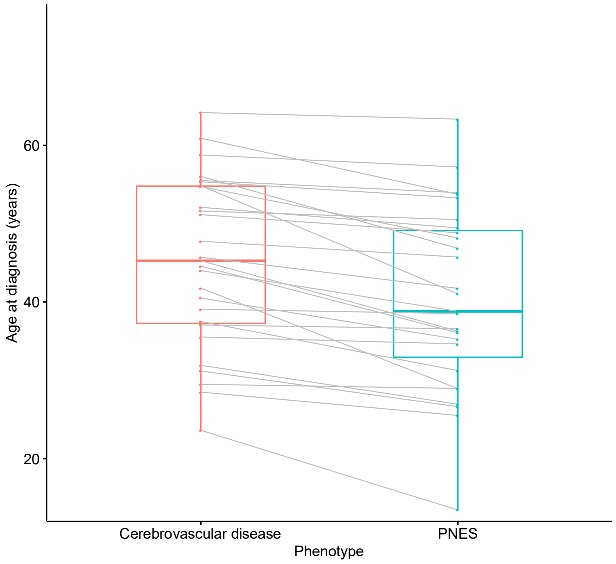

B.

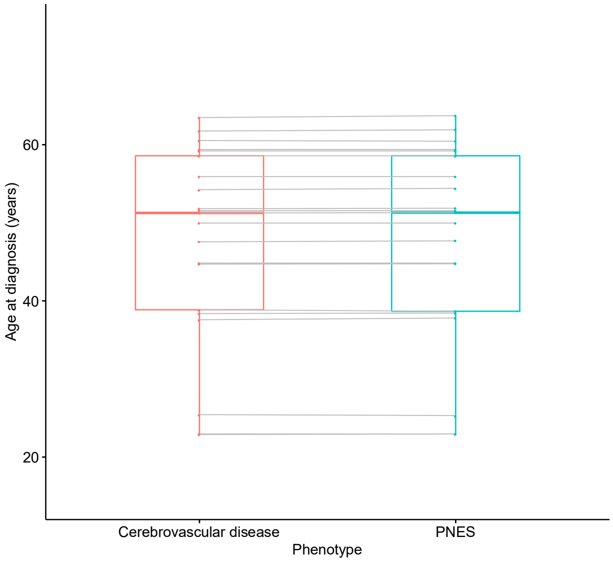

C.

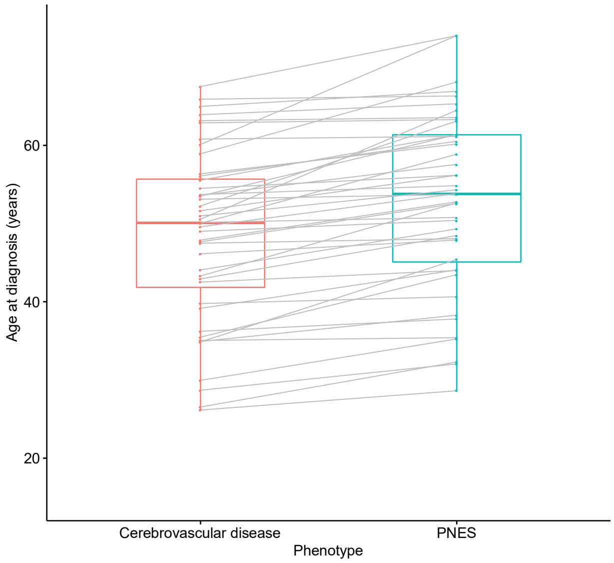

Patients are binned into three groups: A. Patients who developed functional seizures before CVD (n = 27); B. Patients who developed functional seizures and CVD within 3 months of each other (n = 21); and C. Patients who developed CVD before functional seizures (n = 44). The age at the first CVD ICD code each patient was diagnosed with is plotted as red dots, with median and first and third quartiles plotted as a box plot. The corresponding age at functional seizures diagnosis is plotted as blue dots, with a grey line connecting the age of diagnosis of CVD and functional seizures for each patient.

© 2020 Goleva SB et al. *JAMA Network Open.*

eFigure 6. Functional seizures patients have greater BMIs than the control group and epilepsy patients

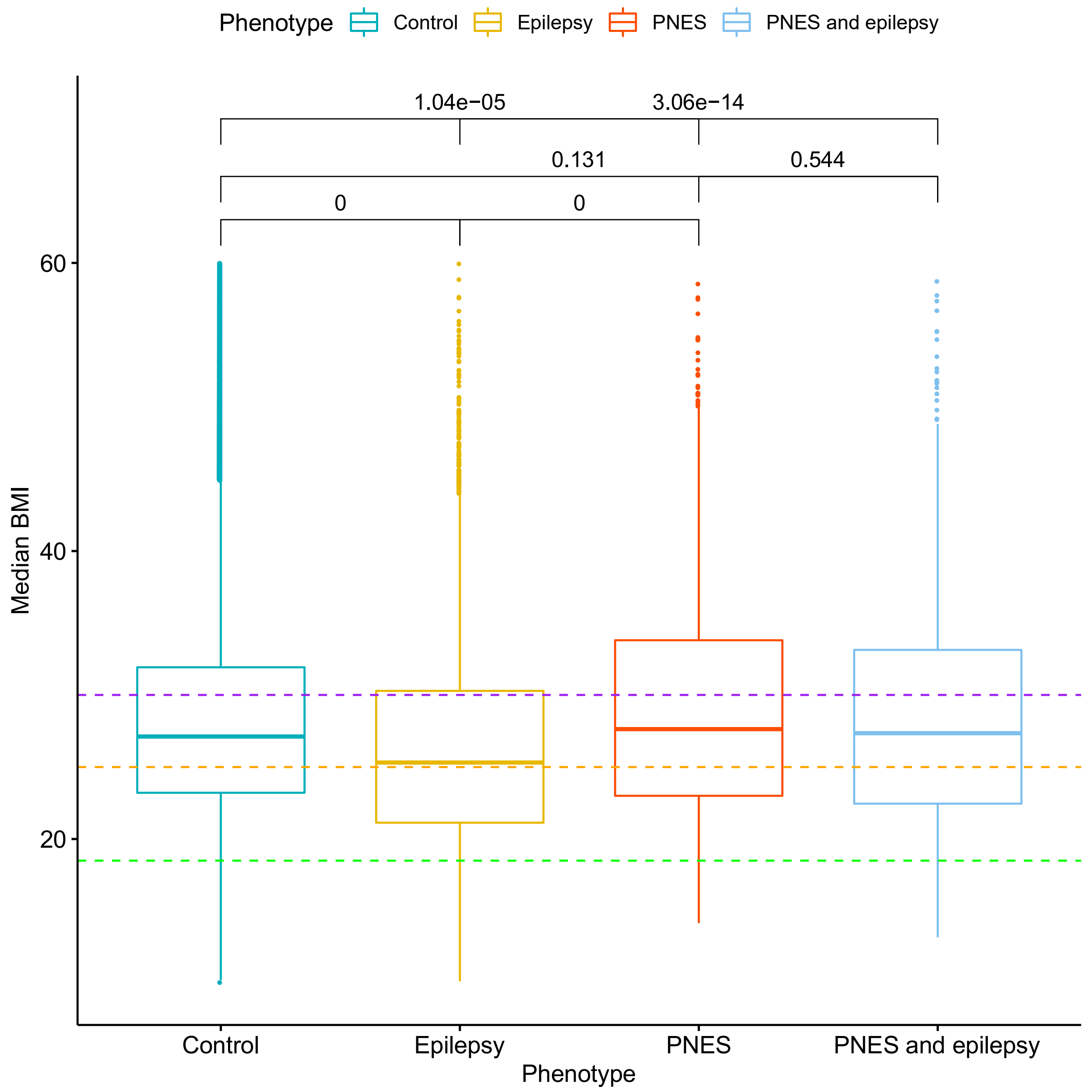

Average and SE of median BMI are shown for controls as identified by our algorithm, epilepsy patients as identified by ICD codes, functional seizures cases as identified by our algorithm, and functional seizures cases with epilepsy as identified by our second algorithm. All participants analyzed had a median age of records over 18. The sample size and percentage of patients from each group that were considered obese (BMI over 30) is also shown.

© 2020 Goleva SB et al. *JAMA Network Open.*

eReferences

1. Jette N, Beghi E, Hesdorffer D, et al. ICD coding for epilepsy: Past, present, and future—A report by the International League Against Epilepsy Task Force on ICD codes in epilepsy. *Epilepsia* 2015; 56(3): 348–55.
2. LaFrance WC, Baker GA, Duncan R, Goldstein LH, Reuber M. Minimum requirements for the diagnosis of psychogenic nonepileptic seizures: A staged approach. *Epilepsia* 2013; 54(11): 2005–18.
3. Tingley D, Yamamoto T, Hirose K, Keele L, Imai K. mediation: R package for causal mediation analysis. *J Stat Software* 2014; 59(5)
4. Leu C, Stevelink R, Smith AW, et al. Polygenic burden in focal and generalized epilepsies. *Brain* 2019; 142(11): 3473–81.
5. Goodloe R, Farber-Eger E, Boston J, Crawford DC, Bush WS. Reducing Clinical Noise for Body Mass Index Measures Due to Unit and Transcription Errors in the Electronic Health Record. *AMIA Jt Summits Transl Sci Proc* 2017; 2017: 102–11.
6. Marquez AV, Farias ST, Apperson M, et al. Psychogenic nonepileptic seizures are associated with an increased risk of obesity. *Epilepsy & Behavior* 2004; 5(1): 88–93.

© 2020 Goleva SB et al. *JAMA Network Open.*
